# Lymphocyte and Platelet Counts, as well as Interleukin-6 Levels, Predict Mortality of Coronavirus Disease 2019 (COVID-19): A Systematic Review and Meta-Analysis

**DOI:** 10.1155/2021/5582908

**Published:** 2021-12-20

**Authors:** Huiwei Chen, Guang Yang, Yunzhu Long, Chaoqian Li

**Affiliations:** ^1^Department of Emergency, The First Affiliated Hospital of Guangxi Medical University, 6 Shuangyong Road, Qingxiu District, Nanning, Guangxi 530021, China; ^2^Zhuzhou Central Hospital, ZhuZhou, Hunan 412000, China

## Abstract

**Objective:**

To systematically evaluate the value of lymphocytes, platelets, and interleukin-6 in predicting the mortality of patients with coronavirus disease 2019 (COVID-19) and to provide medical evidence for the long-term prognosis of patients with COVID-19.

**Methods:**

The latest studies published until July 1, 2021, were retrieved from databases including PubMed, Embase, and Cochrane Library to analyze the ability of lymphocyte and platelet counts as well as interleukin-6 levels to predict mortality in patients with COVID-19. Two reviewers independently screened the literature and extracted data, then evaluated the risk of bias of included studies using the Newcastle–Ottawa Scale (NOS), and used Stata 15.0 software for meta-analysis.

**Results:**

A total of nine studies were included, involving 4340 patients. There were 1330 patients in the death group and 3010 patients in the survival group. Meta-analysis showed that, compared with the survival group, lymphocyte counts in the death group were significantly lower (SMD = −0.64, 95% CI: −0.86–−0.43, *p* < 0.01), platelet counts were significantly lower (SMD = −0.47, 95% CI: −0.67–−0.27, *p* < 0.01), and interleukin-6 levels were significantly higher (SMD = 1.07, 95% CI: 0.62–1.53, *p* < 0.01).

**Conclusion:**

Lymphocyte and platelet counts, as well as interleukin-6 levels, can help predict the mortality of patients with COVID-19. Due to the limitation of the number and quality of the included studies, these conclusions need to be validated by additional high-quality studies.

## 1. Introduction

In 2020, COVID-19 has caused a pandemic. At the time of this report, confirmed cases and the number of deaths were continuing to grow. A large number of studies have shown that some laboratory indicators can help predict the severity of the disease. It is recommended to monitor lymphocyte and platelet counts, as well as interleukin-6 levels, as markers of disease progression [1]. However, only a few studies have mentioned the clinical and laboratory parameters related to death in patients with COVID-19, and most of them involved single centers with small samples. Knowledge of particular laboratory indicators and data that could predict mortality would assist medical personnel in optimal allocation of medical resources so as to provide appropriate interventions in a timely fashion, hopefully reducing mortality. Therefore, we conducted a meta-analysis to determine the role of lymphocyte and platelet counts as well as interleukin-6 levels as predictors of death in patients with COVID-19.

## 2. Materials and Methods

### 2.1. Inclusion and Exclusion Criteria

The inclusion criteria were as follows: (1) definitive diagnosis with COVID-19 and no limitations regarding gender, ethnicity, or nationality; (2) controlled trials and observational studies involving a death group and a survival group; and (3) sample of cases was more than 50. The exclusion criteria were as follows: (1) case reports, abstracts, meta-analyses, reviews, or animal experiments; (2) repetitive studies published by the same author and the same unit; (3) studies involving pregnant women, infants, and children; and (4) incomplete or unusable data, and these data could not be acquired by contacting the author.

### 2.2. Search Strategy

We carried out a systematic electronic literature search of PubMed, Embase, and Cochrane Library. We considered studies published until July 1, 2021, without date or language restrictions, using the following keywords: “lymphocytes,” “lymphocyte,” “Lymphoid Cells,” “Cell, Lymphoid,” “Cells, Lymphoid,” “Lymphoid Cell,” “Blood Platelet,” “Platelet, Blood,” “Platelets, Blood,” “Thrombocytes,” “Thrombocyte,” “Platelets,” “Platelet,” “Interleukin 6,” “IL6,” “Interleukin-6,” “IL-6,” “COVID-19,” “COVID-2019,” “2019nCoV,” “2019-nCoV,” “severe acute respiratory syndrome coronavirus 2,” “SARS-CoV-2,” “2019 novel coronavirus disease,” and “coronavirus disease 2019.” Gray literature was searched as suggested in the current Cochrane Collaboration guidelines, using gray literature databases. Simultaneously, we looked up relevant studies by referencing other studies. Relevant references cited in the retrieved works were also screened when they were considered potentially pertinent.  Table 1 indicates the Cochrane PICO search criteria for our meta-analysis. The study selection process is summarized in Table 2.

### 2.3. Data Collection

Two researchers independently searched the literature and reviewed all titles and abstracts for eligibility. Then, they independently evaluated full texts for inclusion, resolving any disagreement by discussion. The extracted data included the following items: first author's name, country of origin, publication year, study design, sample size, gender, and the count of lymphocytes, platelets, and interleukin-6 of two groups. According to Luo et al. and Wan et al., the mean and standard deviation can be extrapolated from sample size, median, and interquartile range (IQR) when the data in the literature cannot be used directly [2, 3].

### 2.4. Quality Assessment for Research Inclusion

The methodological quality of the included studies was independently assessed by two researchers using the Newcastle–Ottawa Scale (NOS) with some modifications to match this study's needs. The NOS is widely used for the qualitative evaluation of nonrandomized studies in three domains: patient selection, comparability, and results' assessments of the research participants. The highest score is 9 points. Scores more than 6 points indicate high-quality methodology (Table 3).

### 2.5. Statistical Analysis

Stata 15.0 software was used to conduct a meta-analysis of the data. Publication bias was estimated based on the inspection of funnel plots. Effect size was expressed as weighted mean difference (WMD) and 95% confidence interval (CI). Studies with an *I*^2^ statistic >50% or *p* < 0.1 were considered to have significant heterogeneity. A fixed-effect model was used if there was no significant heterogeneity. Otherwise, a random-effect model was chosen.

## 3. Results and Discussion

### 3.1. Results

We identified 985 references after an initial search. After strict screening using inclusion and exclusion criteria, eight retrospective studies [4–9] and a prospective study [10] were included, with a total of 4340 patients. The literature screening process and results are shown in Table 2. The baseline characteristics are given in Table 4.

### 3.2. Meta-Analysis Results

**Figure 1:**
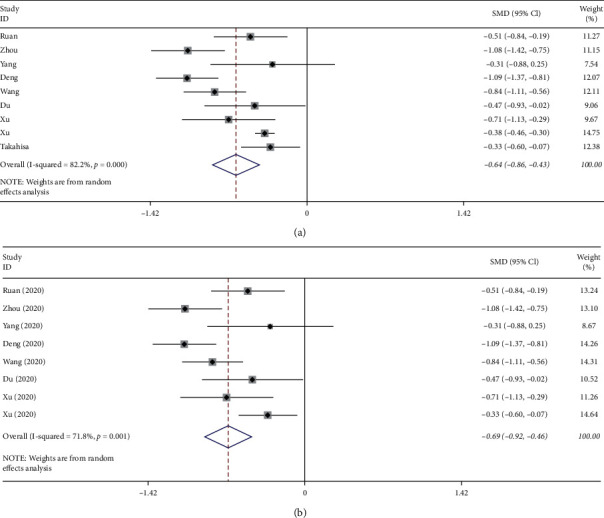
(a) Overall forest plot of lymphocyte counts predicting mortality of COVID-19. (b) Forest plot of lymphocyte counts predicting mortality of COVID-19 after Takahisa et al. dropout.

Nine studies [4–12] provided lymphocyte counts in the death and survival groups; the results of meta-analysis are shown in Figure 1(a). There was significant heterogeneity (*p* ≤ 0.01, *I*^2^ = 82.2%) among studies; therefore, a random-effect model was adopted to conduct a synthesized analysis of the data. This showed that lymphocyte counts in the death group were significantly lower than those of the survival group (SMD = −0.64, 95% CI: −0.86–−0.43).

**Figure 2:**
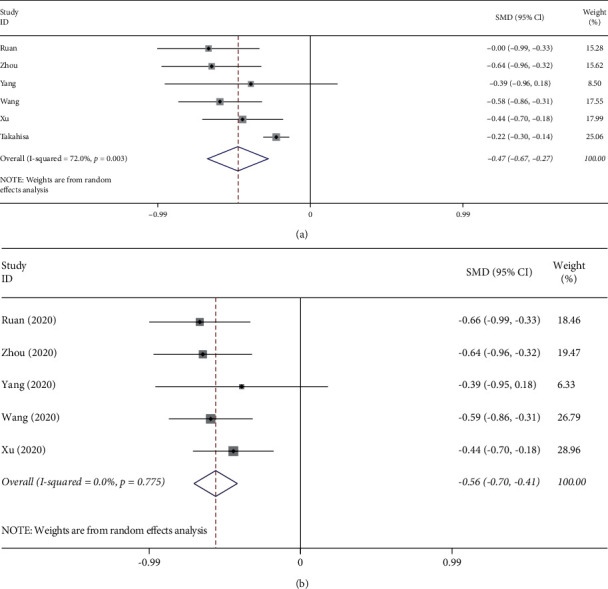
(a) Overall forest plot of platelet counts predicting mortality of COVID-19. (b) Forest plot of platelet counts predicting mortality of COVID-19 after Takahisa et al. dropout.

Six studies [4–6, 8, 11, 12] provided platelet counts in the death and survival groups, and the results of meta-analysis are shown in Figure 2(a). According to the heterogeneity test results (*p* ≤ 0.01, *I*^2^ = 72%), there was significant heterogeneity among studies, and a random-effect model was used to calculate the SMD and 95% CI. This showed that platelet counts in the death group were significantly lower than those of the survival group (SMD = −0.47, 95% CI: −0.67–−0.27).

**Figure 3:**
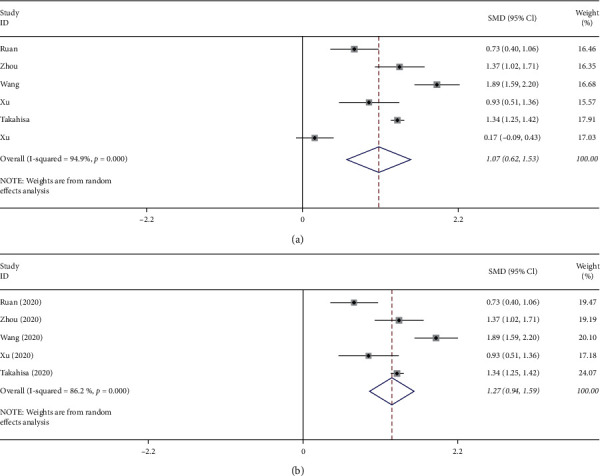
(a) Overall forest plot of interleukin-6 levels predicting mortality of COVID-19. (b) Forest plot of interleukin-6 levels predicting mortality of COVID-19 after Xu et al. dropout.

Six studies [4, 5, 8, 9, 11, 12] provided interleukin-6 levels, and the results of meta-analysis are shown in Figure 3(a). Because of the significant heterogeneity among the studies (*p* ≤ 0.01, *I*^2^ = 94.9%), a random-effect model was used to conduct the analysis. This demonstrated that interleukin-6 levels in the death group were significantly higher than those of the survival group (SMD = 1.07, 95% CI: 0.62–1.53).

### 3.3. Sensitivity Analysis and Heterogeneity

**Figure 4:**
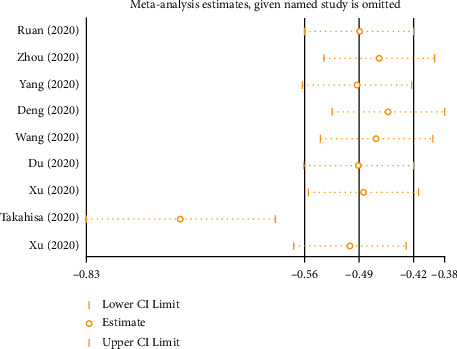
The pooled SMD of sensitivity analyses for the predictive effect of lymphocyte on mortality.

Each study was sequentially removed to evaluate the effect of an individual study on the pooled SMD (Figure 4). The Takahisa et al. study was identified as considerably contributing to the heterogeneity in this pooled effect. After performing sensitivity analysis and after dropping out of Takahisa et al.'s article, the heterogeneity (*I*^2^) of lymphocyte counts in the groups was reduced from 82.2% to 71.8% (Figure 1(b)), and the heterogeneity (*I*^2^) of platelet counts in the groups was reduced from 72% to 0% (Figure 2(b)). After performing sensitivity analysis and after dropping out of Xu et al.'s article, the heterogeneity (*I*^2^) of interleukin-6 levels in the groups was reduced from 94.9% to 86.2% (Figure 3(b)). Because fewer than ten studies were finally involved in this study, causing test efficiency to be insufficient, we did not generate a funnel plot. No subgroup analysis was done because only one study data was from New York, USA, and the rest were from Wuhan, China, and only one study data was a prospective study, the rest were retrospective studies, and only one study data listed IBM data.

### 3.4. Publication Bias Assessment

**Figure 5:**
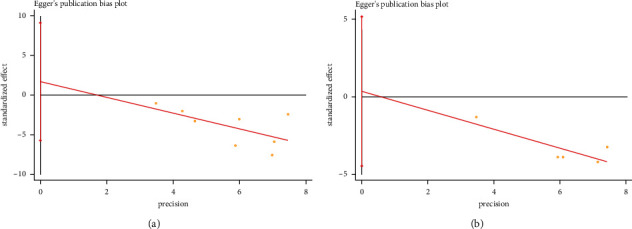
Publication bias assessment by using Egger's test for lymphocyte counts and platelet counts.

Publication bias is defined as the problem that results from systematic differences between the results of all completed studies on a topic and the subset of those studies that are published [13]. Statistical Egger's test indicated that there was no evidence of publication bias for lymphocyte counts (*p*=0.594) and platelet counts (*p*=0.833) in the overall analysis (Figures 5(a) and 5(b)). Thus, it seems that the results of this study were eligible and were not affected by publication bias.

## 4. Discussion

COVID-19 started in December 2019 and swept across the world; it has not yet been controlled. Although most infected people have only mild symptoms, some cases can quickly progress to pneumonia, multiple organ failure, and even death [7]. This new virus has caused global panic and has affected the global economy. To date, there are no specific antiviral drugs, and vaccines remain in the research and trial stage. For these reasons, identification of markers that can predict the severity and death from the disease would facilitate early intervention and treatment.

The lymphocyte count of patients in the death group was significantly lower than that of the survival group, similar to findings from studies on SARS [14]. Studies have shown that persistent lymphocytopenia in sepsis predicts early and late mortality [15, 16]. The degree of lymphocytopenia might reveal either the severity of viral invasion or the state of antiviral immunity, both of which are key factors related to the severity and mortality of various diseases [8]. Lymphocytopenia is a prominent feature of critically ill patients infected with SARS-CoV because targeted invasion by SARS-CoV viral particles destroys the cytoplasmic component of the lymphocyte, leading to its destruction [17]. Lymphocytopenia is a common feature of patients with COVID-19; therefore, we hypothesized that necrosis or apoptosis of lymphocytes would also induce lymphocytopenia in critically ill patients infected with SARS-CoV-2 [6].

In this study, platelet counts in the death group were significantly lower, correlating with in-hospital death. Thrombocytopenia is common in patients with COVID-19, and it is associated with an increased risk of in-hospital mortality. The lower the platelet count, the higher the mortality [18]. In patients with SARS-CoV infection, thrombocytopenia was found in 40% to 50% patients [19–21]. Hematopoiesis can be induced by SARS-CoV after infecting cells in the bone marrow. Because the current coronavirus shares 79% genomic sequence with SARS-CoV and the same cell entry receptor of angiotensin-converting enzyme 2 [22], we speculate that SARS-CoV-2 may cause thrombocytopenia in a similar way.

Interleukin-6 levels increased significantly, positively correlating with in-hospital death. IL-6 is a key mediator regulating inflammation. As is well known, systematic inflammation and oxidative stress are related to high plasma levels of proinflammatory cytokines, such as IL-6 [23]. The increase of IL-6 levels reflects the severity of inflammation and is a feature of “cytokine storm.” In cases of MERS, levels of several inflammatory mediators, including interleukin-6, strongly correlated with mortality [24]. The significant increases in IL-6 levels in the present study suggest that SARS-CoV-2 infection damages the immune system and leads to systemic inflammatory responses [25]. Death cases characterized by significant increases in inflammatory markers may represent more severe inflammatory responses.

A limited number of included studies included variables such as cardiac troponin, C-reactive protein, and serum ferritin, and there were no sufficient data in primary studies. These limitations contributed to our inability to find resolute conclusions in these regards. In addition, another limitation that may have impacted the results of this systematic review was the different type and stage of diseases in primary studies which likely impacted inflammatory marker levels.

On the contrary, most studies included in our study, as well as the previous meta-analysis study, were carried out in China which increase the possibility of selection bias. So, the obtained results may not be applicable to other populations in different geographic areas. The potential heterogeneity was hypothesized as being derived from clinical factors, such as treatment strategy and severity of infection. Unfortunately, when studies are compared in a meta-analysis, it is difficult to provide definitive conclusions about heterogeneity [26].

Because of several limitations, our results should be interpreted cautiously. First, most of the included studies were retrospective, and we did not have sufficient information about study participant characteristics such as BMI, standard of care, or medication regimens. Second, all involved studies were from China. This reduces the strength and generalizability of the results. Furthermore, the reliability of our pooled analysis was affected unavoidably by risk of bias. Nevertheless, this is a rare study of the predictors of death from COVID-19. The meta-analysis gave clear results and demonstrated the value of various mortality predictors. The results of this review may provide early warnings of death in critically ill patients and may facilitate early intervention and treatment.

## 5. Conclusions

Lymphocyte and platelet counts, as well as interleukin-6 levels, can predict mortality of COVID-19 and help clinicians evaluate patient outcomes. Further investigation is needed.

## Figures and Tables

**Table 1:** Description of PICO.

Condition	Description

Participant	Patients with COVID-19
Intervention	COVID-19
Comparison	Nonsurvivor group versus survivor group
Outcome	Lymphocyte and platelet counts and interleukin-6 levels

**Table 2:** The literature screening process and results.

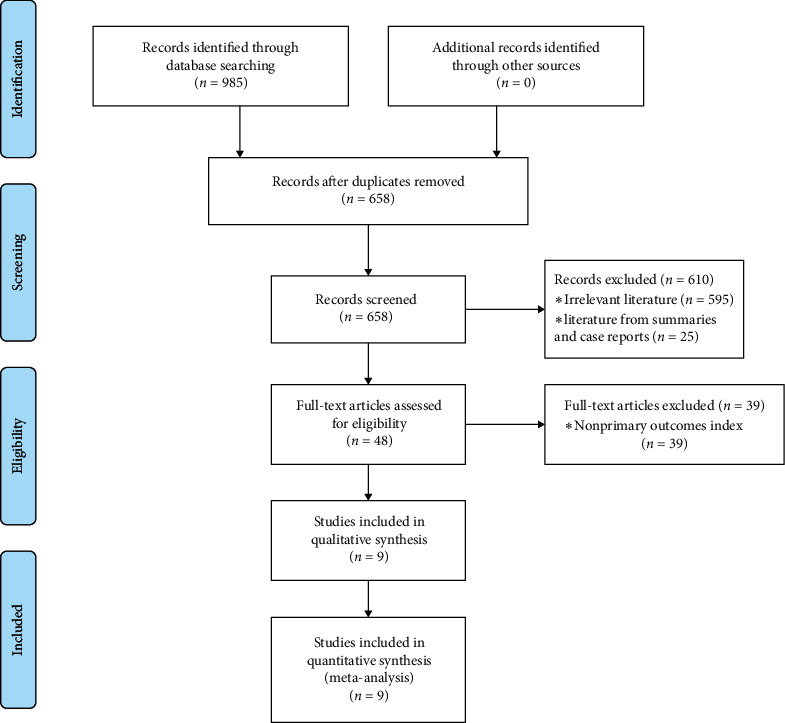

**Table 3:** Methodological quality of enrolled studies based on the Newcastle–Ottawa Scale (NOS).

Study	Is the definition adequate?	Representativeness of the cases	Selection of controls	Definition of controls	Comparability of both groups	Ascertainment of diagnosis	Same ascertainment method for both groups	Nonresponse rate	Total scores

Ruan. Q	★	★	★	—	—	★	★	★	7
Zhou. F	★	★	★	★	★★	★	★	★	9
Yang. X	★	★	★	★	★-	★	★	★	8
Deng. Y	★	★	★	★	—	★	★	★	7
Wang. L	★	★	★	★	★-	★	★	★	8
Xu. B	★	★	—	★	★-	★	★	★	7
Du. R. H	★	★	★	★	—	★	★	★	7
Xu. J. Q	★	★	★	★	★-	★	★	★	8
Takahisa. M	★	★	★	★	—	★	★	★	7

**Table 4:** The baseline characteristics.

Study	Year	Country	Type of study	Sample	Age	Male	Lymphocyte count (×10^9^/L)	Platelet count (×10^9^/L)	IL-6 (pg/mL)
					*Survival*				
Ruan et al. [a]					50 (44–81)	53 (65%)	1.42 (2.14)	222.1 (78.0)	6.8 (3.61)
Zhou et al. [b]	2020	China	Retrospective	191	52 (45–58)	81 (59%)	1.1 (0.8–1.5)	220.0 (168.0–271.0)	6.3 (5·0–7.9)
Yang et al. [c]	2020	China	Retrospective	52	51.9 (12.9)	14 (70%)	0.74 (0.40)	164 (74)	
Deng et al. [d]	2020	China	Retrospective	225	40 (33, 57)	51 (44%)	1.00 (0.72, 1.27)		
Wang et al. [e]	2020	China	Retrospective	339	68 (64–74)	127 (49%)	0.97 (0.68–1.37)	211 (159–268)	10.5 (4.9–18.8)
Du et al. [f]	2020	China	Prospective	179	56.0 ± 13.5	87 (55.1%)	0.8 (0.6–1.1)		
Xu et al. [g]	2020	China	Retrospective	145	56 [43, 66]	59 (50.4%)	0.93 [0.65, 1.37]		14.65 [4.24, 27]
Takahisa et al. [h]	2020	USA	Retrospective	2820	62 [49, 73]	1128/2014 (56%)	0.90 [0.70, 1.30]	212.0 [166.0, 267.0]	45.8 [23.3, 82.4]
Xu et al. [i]	2020	China	Retrospective	239	57.5 ± 13.5	53 (57.6%)	0.7 [0.50–0.9]	186 [148–232]	9.1 [6.2–11.7]

					*Death*				
Ruan et al. [a]	2020	China	Retrospective	150	67 (15–81)	49 (72%)	0.60 (0.32)	173.6 (67.7)	11.4 (8.5)
Zhou et al. [b]	2020	China	Retrospective	191	69 (63–76)	38 (70%)	0.6 (0.5–0.8)	165.5 (107.0–229.0)	11.0 (7.5–14.4)
Yang et al. [c]	2020	China	Retrospective	52	64.6 (11.2)	21 (66%)	0.62 (0.37)	191 (63)	
Deng et al. [d]	2020	China	Retrospective	225	69 (62, 74)	73 (67.0)	0.63 (0.40, 0.79)		
Wang et al. [e]	2020	China	Retrospective	339	76 (70–83)	39 (60%)	0.57 (0.39–0.84)	172 (103–219)	93.8 (35.9–182.3)
Du et al. [f]	2020	China	Prospective	179	70.2 ± 7.7	10 (47.6)	0.7 (0.5–0.8)		
Xu et al. [g]	2020	China	Retrospective	145	73 [68, 77.25]	17 (60.7)	0.56 [0.32, 0.94]		29.8 [14.6, 63.89]
Takahisa et al. [h]	2020	USA	Retrospective	2820	76 [65, 85]	483/806 (59.9)	0.80 [0.50, 1.10]	197.0 [146.0, 252.0]	152.4 [79.1, 303.8]
Xu et al. [i]	2020	China	Retrospective	239	65.7 ± 12.2	90 (61.2%)	0.6 [0.4–0.8]	160 [110–206]	9.1 [7.1–12.9]

## Data Availability

The data used to support the findings of this study are available from the corresponding author upon request.
